# The Evidence for a Causal Link Between Disease and Damaging Behavior in Pigs

**DOI:** 10.3389/fvets.2021.771682

**Published:** 2022-01-27

**Authors:** Laura A. Boyle, Sandra A. Edwards, J. Elizabeth Bolhuis, Françoise Pol, Manja Zupan Šemrov, Sabine Schütze, Janicke Nordgreen, Nadya Bozakova, Evangelia N. Sossidou, Anna Valros

**Affiliations:** ^1^Teagasc Animal and Grassland Research and Innovation Centre, Cork, Ireland; ^2^School of Natural and Environmental Sciences, Newcastle University, Newcastle upon Tyne, United Kingdom; ^3^Adaptation Physiology Group, Department of Animal Sciences, Wageningen University & Research, Wageningen, Netherlands; ^4^ONIRIS, Nantes, France; ^5^Biotechnical Faculty, Department of Animal Science, University of Ljubljana, Ljubljana, Slovenia; ^6^Chamber of Agriculture of North Rhine-Westphalia, Animal Health Services, Bad Sassendorf, Germany; ^7^Faculty of Veterinary Medicine, Department of Paraclinical Sciences, Norwegian University of Life Sciences, Oslo, Norway; ^8^Faculty of Veterinary Medicine, Trakia University, Stara Zagora, Bulgaria; ^9^Ellinikos Georgikos Organismos-DIMITRA (ELGO-DIMITRA), Veterinary Research Institute, Thessaloniki, Greece; ^10^Department of Production Animal Medicine, Research Centre for Animal Welfare, University of Helsinki, Helsinki, Finland

**Keywords:** welfare, health, lesion, risk, behavior, pig, tail biting

## Abstract

Damaging behaviors (DB) such as tail and ear biting are prevalent in pig production and reduce welfare and performance. Anecdotal reports suggest that health challenges increase the risk of tail-biting. The prevalence of tail damage and health problems show high correlations across batches within and between farms. There are many common risk factors for tail-biting and health problems, notably respiratory, enteric and locomotory diseases. These include suboptimal thermal climate, hygiene, stocking density and feed quality. The prevalence of tail damage and health problems also show high correlations across batches within and between farms. However, limited evidence supports two likely causal mechanisms for a direct link between DB and health problems. The first is that generalized poor health (e.g., enzootic pneumonia) on farm poses an increased risk of pigs performing DB. Recent studies indicate a possible causal link between an experimental inflammation and an increase in DB, and suggest a link between cytokines and tail-biting. The negative effects of poor health on the ingestion and processing of nutrients means that immune-stimulated pigs may develop specific nutrient deficiencies, increasing DB. The second causal mechanism involves tail-biting causing poor health. Indirectly, pathogens enter the body via the tail lesion and once infected, systemic spread of infection may occur. This occurs mainly via the venous route targeting the lungs, and to a lesser extent via cerebrospinal fluid and the lymphatic system. In carcasses with tail lesions, there is an increase in lung lesions, abscessation, arthritis and osteomyelitis. There is also evidence for the direct spread of pathogens between biters and victims. In summary, the literature supports the association between poor health and DB, particularly tail-biting. However, there is insufficient evidence to confirm causality in either direction. Nevertheless, the limited evidence is compelling enough to suggest that improvements to management and housing to enhance pig health will reduce DB. In the same way, improvements to housing and management designed to address DB, are likely to result in benefits to pig health. While most of the available literature relates to tail-biting, we suggest that similar mechanisms are responsible for links between health and other DB.

## Introduction

The health and welfare of intensively farmed pigs is an important topic of research for decades. Currently the drivers for work in this area include (1) the threat of antimicrobial resistance and the associated mis/overuse of antibiotics in animal production, (2) unprecedented levels of societal concern and interest in animal production practices (3) and the related renewed impetus to implement legislation on animal welfare in the EU [e.g., on tail docking—(1)]. These share a cross cutting theme of the interplay between animal health and animal welfare, which is a growing area of research interest [e.g., (2)]. In this review animal health is defined as the absence of disease in the main physiological (i.e., respiratory, enteric and locomotory) systems and animal welfare is defined as “the physical and mental state of an animal in relation to the conditions in which it lives and dies” (3).

Damaging behaviors (DB) involve oral manipulation of a body part of another pig with the outcome being tissue damage at the targeted body part (referred to as “lesions” in this paper, unless otherwise indicated). DB include tail, ear and flank biting (4–6) with leg and penis biting also reported (7), albeit much less commonly. DB are commonly performed by growing pigs but sows also perform vulva biting (8). Furthermore, there are cases of piglets directing DB toward sows in the farrowing crate (9, 10). Only DB performed by grower-finisher pigs will be considered in this review. Furthermore, although aggression also causes lesions (11) it is not included as it is not considered a DB but forms a normal part of the agonistic behavioral repertoire of the pig (12).

The high prevalence and widespread nature of tail-biting and the contentious way it is controlled (i.e., tail docking) means that it is the most widely studied of all DB [e.g., (13–16)]. Whilst most prevalent in intensive housing systems, tail lesions, usually attributed to tail biting behavior, are also found in straw based (17) and free range/organic production systems (18–20). Tail-biting has negative economic implications as it increases days to slaughter (21) due to reduced growth rates (22, 23), and is related to poor carcass characteristics and an increased risk of carcass condemnation (24–26). It is associated with poor welfare because of the stress and the pain experienced by the bitten pig (27, 28). Such lesions cause additional suffering because of the risk of systemic spread of infection to various organs including the lungs (29–31). There is also some evidence suggesting that pigs performing tail-biting are suffering from stress (28).

The reported incidence and prevalence of tail-biting varies widely between and within farms (25, 32), reflecting the multifactorial etiology of tail-biting (33) and possibly all DB. The risk of tail-biting is increased by management and housing practices that fail to meet the basic needs of pigs, thereby causing stress (28, 34). In addition, acute stress (e.g., caused by a blocked feeder) may cause a sudden outbreak of DB even on a farm where the tail-biting risk is otherwise well-managed (35). Management and housing practices present numerous risk factors relating to diet, regrouping strategy, group composition, space allowance, climate and enrichment. These interact with animal factors such as genetics, sex, age/weight and health. As risk factors differ for every farm, a farm specific solution is required, thereby complicating the development of more generalized prevention strategies (13). Further complication arises from the fact that there are at least three different types of tail-biting, which may have a different motivational background and therefore potentially different risk factors (33).

Ear lesions are a growing problem on pig farms, with a recent study reporting a 100% farm level prevalence on 31 farms with a median of 6.97% animals affected (16). Ear lesions result from factors that compromise skin defenses, allowing the entry of infective agents [e.g., (36)]. High levels of oral behavior (nibbling and chewing) directed toward the ears (37) suggest that, in some cases, DB is the cause of ear lesions. This etiology is well-accepted for tail lesions, where an outbreak of tail-biting is often preceded by a period of tail manipulation without any injured tails (38) and once the tails are damaged and blood is present the problem escalates (39, 40). However, certainty about the etiology of ear lesions is complicated by the relative lack of research on ear biting behavior and on related conditions such as ear necrosis (“dead ear tissue”). Furthermore, similar to tail-biting, there may be three different types of ear biting (37) which further complicates the issue.

Different forms of DB may have some causal factors in common or indeed may be causally linked. Several studies report that individual animals performing more tail-biting also perform more of other DB [e.g., (4, 10)], and treatments that influence tail-biting often influence ear biting and other DB [e.g., (10, 41–43)]. Furthermore, on farms where tails are docked short there is more ear biting (44). Taken together this suggests that at least part of the animal- and environment-based risk factors may overlap for tail-biting and the “less-studied” DB.

There is much anecdotal evidence supporting the putative role that health plays in DB (45, 46). Indeed, disease and tail lesion prevalence correlate at the farm (14) and abattoir (47) level. This may be related to the commonality of risk factors between DB and health problems, for example the absence of straw is associated with a higher risk of tail biting and lameness (48). However, evidence of direct causal links also exist. Animals that feel unwell and/or stressed, experience an immune reaction and altered metabolic state (49), and this may lead them to increase manipulatory behavior of their penmates (50–52). On the other hand, sickness also causes pigs to respond less to general manipulation by their pen mates, thereby placing them at higher risk of injury (51, 52). The potential mechanism for the role sickness plays in DB (52) was explored recently (53). The objective of the current paper is to review links between health and DB in pigs, to assess the evidence for a causal relationship in both directions and to elucidate potential mechanistic pathways.

## Correlations Between the Prevalence of Disease or Lesions and Damaging Behavior

The first evidence for links between health and DB comes from studies showing correlations between disease or disease lesions and DB or the associated lesions at farm and abattoir level. Studies describing such associations almost all focus on tail lesions. There are few studies showing links between ear lesions and disease other than those showing the obvious association between ear lesions and ear necrosis [see Malika et al. for review (54)]. Though recently Pessoa et al. (55) reported an association between ear lesions during the grower phase and pericarditis at slaughter. They suggest possible mediation via *Streptococcus suis* in the saliva. Schroder-Petersen and Simonsen (38) also cite a Dutch study by Elst et al. (56) in which a correlation of 0.25 was found between the percentage of weaned piglets with respiratory disease and ear (and tail) biting problems at farm level.

### Tail Data From Live Animals On-Farm and Disease Data

At farm level, authors report an association (14) or a tendency for one between the presence of respiratory diseases and tail-biting (57). Furthermore, Pandolfi et al. (58) showed that the prevalence of severe tail-biting on farms was associated with the prevalence of enzootic pneumonia-like lesions at slaughter. In a controlled farm and abattoir-based study, Marques et al. (59) found an association between tail lesions measured on farm and post-mortem lung lesions (pleuritis and embolic pneumonia). However, at individual pig level the study found no association between tail lesions and new cases of respiratory disease on farm. Indeed, it is difficult to detect respiratory disease in individual pigs in a group on-farm and it often goes unnoticed. Also at individual level, Marques et al. (59) reported that pigs with severe tail lesions had higher odds of presenting locomotion disorders than pigs with no lesion. Similarly, Vom Brocke et al. (60) found a correlation between tail lesions of any severity and leg inflammation and Niemi et al. (61) found more lameness in victims of tail-biting (20% affected) compared to non-victims (9% affected). Interestingly, in the study by Niemi et al. (61) lameness was diagnosed 3.7 days before the pig was diagnosed as a victim of tail-biting suggesting a causal relationship. These authors also reported a 1.8-fold higher risk of generalized health disorders in victims of tail-biting compared to non-victims.

### Tail and Disease Data Collected at the Abattoir

As with live animals, data are available on correlations between disease lesions and tail lesions in the slaughtered pig (Table 1). Observed lesions are localized on viscera, mainly reflecting pathologies in the respiratory system or on the carcass (mainly arthritis or abscessation). Potential outcomes of the latter include trimming and partial or entire condemnation. Potentially important associations between tail lesions and disorders of other systems e.g., the digestive tract, cannot be evaluated at the abattoir because the intestines are not routinely inspected.

**Table 1:** Overview of studies showing correlations between tail and ear lesions on farm or at slaughter and carcass findings at slaughter.

**References**	**Place of observation of tail or ear damage**	**Sample**	**Observed tail or ear lesions**	**Level of analysis**	**Localization and type of correlated lesions or outcome**	**Type of lesions observed but not correlated**
Elbers et al. (62)	At slaughter (2 abattoirs) The Netherlands	550,000 pigs from 205 herds	Inflammatory changes of tail (as thickening) (presence vs. absence)	Herd	Lung abscesses Severe pleurisy Pneumonia Arthritis Atrophic rhinitis Inflammation of the legs	Skin lesions Pleurisy Lesions in liver
ter Elst-Whale et al. (56)	Data collected from a questionnaire among farmers and their vets The Netherlands	Weaned piglets from 438 farms (417 farrow-to-finish farms and 17 rearing farms)	Ear and tail biting	Farm	Bronchial tube problems	Arthritis Meningitis Diarrhea Oedema disease Swing disease
Flesjå and Ulvesaeter (63)	At slaughter (1 abattoir) Norway	354,342 pigs slaughtered between 1974 and 1977	Not defined, but an earlier paper is referred to explain the abattoir scoring system	Animal	Pyemia Abscesses Polyarthritis Arthritis Severe and moderate pneumonia Anemia Vertebral osteomyelitis	Atrophic rhinitis Pleurisy Pericarditis Tuberculous lesions in the cervical lymph nodes Scabies
Harley et al. (24)	At slaughter (6 abattoirs) Republic and Northern Ireland	36,963 pigs from 221 farms; 250 batches	Mild lesions of tail (K&M 2004^a^)	Batch	Entire and partial (hindquarter and/or forequarter) carcase condemnation, due to abscessation, arthritis, pleurisy, pneumonia, peritonitis, pericarditis, pyaemia, scepticaemia, toxemia, bruising, haematoma	
			Severe lesions of tail (K&M 2004^a^)	Batch		Entire and partial (hindquarter and/or forequarter) carcase condemnation, due to: Abscessation Arthritis Pleurisy Pneumonia Peritonitis Pericarditis Pyaemia Scepticaemia Toxemia Bruising Haematoma
Harley et al. (64)	At slaughter (1 abattoir) Ireland	3,422 pigs from 49 farms, 74 batches	Severe lesions of tail (K&M 2004^a^)	Animal	Carcass condemnation and trimming	Condemnation due to abscessation
Huey (30)	At slaughter (1 abattoir) Northern Ireland	75,130 pigs	Abscess on tail (visible)	Animal	Abscesses in lungs, peritoneum, vertebrae, legs Vertebral osteomyelitis	Abscesses on head
Kritas and Morrison (65), Study 2	At slaughter (1 abattoir) Greece	256 pigs (128 bitten pigs and 128 controls)	Mildly and severely bitten tail (K&M 2004^a^)	Animal	Lungs abscesses Pleuritic lesions Carcass abscesses: condemnation (entire, partial) or trimming	Enzootic pneumonia
Marques et al. (59)	On-farm Brazil	312 pigs from 4 farms (104 bitten pigs and 208 controls)	Severe lesion of tail [score 3, Marques et al. (59)^b^]	Animal	Locomotor disorders	Respiratory disorders
			Lesions on tail [scores 1 to 3, Marques et al. (59)^b^]	Animal	Nodules and/or abscesses	
	At slaughter (1 abattoir) Brazil	312 pigs from 4 farms (104 bitten pigs and 208 controls)	Lesions on tail [scores 1 to 3, Marques et al. (59)^b^]	Animal	Abscesses, lung lesions	Arthritis, other lesions
Martinez et al. (66)	At slaughter (1 abattoir) Spain	6,017 pigs	Lesions on tail (binary, present or absent)	Animal	Arthritis Vertebral osteomyelitis	Abscesses
Meijer et al. (67) cited by Huey (30)	At slaughter The Netherlands		Healed or inflammatory tail, degrees of severity of tail lesions		Osteomyelitis (healed tail) Embolic pneumonia (inflammatory tail, not healed)	Embolic pneumonia Osteomyelitis of the vertebrae Abscesses
Moinard et al. (14)	Farm England	92 farms	Presence or absence of tail biting outbreak	Farm	Respiratory diseases Rectal prolapse	Bone disease Alimentary disease
Munsterhjelm et al. (51)	Experimental facilities Norway	95 pigs in experimental facilities (13 with respiratory disease, 37 controls and 45 with other health disorders)	Ears and tail biting (behavioral observation: taking the tail or the ears of another pig in the mouth followed by an immediate reaction by the receiver.)	Animal	Subclinical respiratory disease Respiratory inflammations	Osteochondrosis
Niemi et al. (61)	Farm Finland	6,812 pigs from 1 farm	Tails with visible wounds	Animal	Leg disorder Health disorders	
Pandolfi et al. (58)	On-farm and at slaughter Great Britain	157,887 from 40 fattening farms	Severe lesion on tail [Pandolfi et al. (58)^c^]	Herd	Enzootic pneumonia Lameness Pyaemia	Pleurisy Pericarditis Peritonitis Milk spot Hepatic scarring Papular dermatitis Pleuropneumonia Abscess
			Tail-bitten^d^		Papular dermatitis Pyaemia Peritonitis Abscesses	Lameness Enzootic pneumonia Pericarditis Milk spot Hepatic scarring Pleuropneumonia
Pessoa et al. (55)	Farm and slaughter Ireland	1,573 pigs from 1 farm longitudinal study	Ear lesions (partial or total loss of one or both ears)		Pericarditis	Lameness Bursitis Lung lesions (pneumonia, pleurisy)
Sanchez-Vazquez et al. (68)	17 abattoirs Great Britain	324,250 pigs from 1,138 farms, 6,485 batches	Lesions on tail (binary, present or absent)	Batch	Pyaemia (lung lesions)	Enzootic-pneumonia-like Pleurisy Milk spots Hepatic scarring Pericarditis Peritonitis Abscess (lung) Papular dermatitis
Scollo et al. (57)	Farm Italy	201,790 pigs from 67 heavy pig production farms	Lesions on tail (binary, present or absent) and presence or absence of at least one case of tail biting in the farm	Farm	Respiratory disorders	Enteric disorders
Sihvo et al. (69)	Necropsy examination	36 growing pigs	Severe tail damage	Animal	Chronic purulent or necrotizing interstitial or bronchopneumonia with or without abscesses	Pulmonary actinobacillosis Mild lymphocytic interstitial pneumonia Mild lymphocytic infiltration
Teixeira et al. (47)	At slaughter (1 abattoir) Ireland	3,143 pigs from 36 farms, 61 batches	Mildly and severely bitten tail (K&M 2004^a^)	Animal		Pleurisy, pneumonia, and pleuropneumonia, abscessation, pericarditis, ascariasis
				Batch	Pleurisy, pneumonia, and pleuropneumonia	
Valros et al. (25)	At slaughter (1 abattoir) Finland	10,852 pigs from 479 farms	Presence of lesions on tail (healed, fresh or severe, e.g., fresh and short tail, vs. absent)	Animal	Condemnation for abscessation and arthritis	Bone fractures
Valros et al. (26)	At slaughter (1 abattoir) Finland	14,382 pigs	Presence of lesions (healed, acute (bite marks, minor wounds, major wounds), length of remaining tail.	Animal	Partail and whole carcass condemnations, Abscessed, arthritis, pericarditis, pleuritis, pneumonia, skin infections	Organ condemnation
Van Staaveren et al. (70)	At slaughter (1 abattoir) Ireland	5,628 pigs from 26 farms, 38 batches	Severely bitten tail (K&M 2004^a^)	Animal	Severe pleurisy (tendency)	Pneumonia, abscess, pleuropneumonia
			Any lesion on tail	Batch		Pleurisy, pneumonia, abscess, pleuropneumonia
Vom Brocke et al. (60)	On-farm and at slaughter (1 abattoir) Germany	79,954 pigs from 64 farms	Any lesion^e^ on tail	Animal	Leg inflammation, arthritis and abscesses	Pleurisy
			Severe lesion^e^ on tail	Animal	Lung findings, arthritis and abscesses	
			Tail necrosis^e^	Animal	Pleurisy, lung findings, leg inflammation, arthritis and abscesses	
Walker and Bilkei (20)	On-farm and at slaughter (1 abattoir) Croatia	1,454 pigs from 5 farms	Severely bitten tail (K&M 2004^a^)	Animal	Carcass condemnation	
Wallgren and Lindahl (23)	On-farm and at slaughter (1 abattoir) Sweden	48 pigs from 1 farm	Absence, mild, severe lesions of tail	Animal	Abscesses	Pneumonia, pleuritis, liver condemnation, arthritis

a *Tail lesion scoring system according to Kritas and Morrison (71). 0 No evidence of tail biting, 1 Healed or mild lesions, 2 Evidence of chewing or puncture wounds, but no evidence of swelling, 3 Evidence of chewing or puncture wounds with swelling and signs of possible infection, 4 Partial or total loss of the tail. Scores of 1 and 2 are combined into one category, designated as “mildly bitten carcases,” and those with tail scores of 3 and 4 are combined into another category, designated as “severely bitten carcases.”*

b *Tail lesion scoring system from Marques et al. (59). 0—Without lesion, normal tail; 1—Discrete lesion, with superficial loss of epithelial tissue; 2—Moderate lesion, with up to 50% of the tail injured or lost; 3—Severe lesion, more than 50% of the tail injured or total loss of the tail; 4—Healed lesion*

c *Tail lesion scoring system from “Real Welfare” scheme (72). No lesions—Pigs without any of the above lesions; Mild—Pigs with mild tail lesions; Severe—Pigs with severe tail lesions. Proportion of tail has been removed by biting or tail is swollen or held oddly, or scab covering whole tip or fresh blood visible*.

d *Tail lesion scoring system from “British Pig Health Scheme data.”*

e *Tail lesions scoring system from Vom Brocke et al. (60). Scoring from picture of carcasses: 0—no visible lesion; 1—mild lesion: 2—severe lesion; 3—necrosis; CL—complete loss of tail. Score 0 and CL absent are combined into one category, designated “no lesion”; scores 1, 2 or 3 and/or CL “any lesion”; score 2 or 3 and/or CL—“severe damage.” Scoring from direct meat inspection: tail necrosis—presence/absence*.

Studies look at associations between the presence or absence [e.g., (59)] or severity [e.g., (65)] of tail lesions and viscera and/or carcass lesions in the slaughterhouse. The latter data are either routinely collected as part of meat inspection [e.g., (47)] or involve more detailed measurements using lesion scoring schemes such as that developed by BPEX [e.g., (70)]. Studies based on official meat inspection data are difficult to compare because of inconsistencies between meat inspectors (47, 62) between slaughterhouses (73) and between countries (24, 74). Standardized scoring systems also differ between studies and definitions used of both lung and tail lesions are often vague.

#### Respiratory Lesions

Numerous studies describe an association at herd or batch level between tail lesions and different lesions of the respiratory tract; pleurisy (47, 62), pneumonia (47, 58, 62), and lung abscesses (58, 62, 68) (Table 1). In contrast, van Staaveren et al. (70) did not find a batch-level association between any lesions of the respiratory tract and tail lesions.

The three studies (47, 58, 62) showing batch or herd level associations between lesions related to (enzootic) pneumonia and tail lesions suggest that generalized poor respiratory health on farm might increase the risk of pigs performing biting behavior. Studies showing that pigs with subclinical respiratory disease were more prone to bite the ears and tails support this (51). This might also explain why there is no association between lesions related to pneumonia and tail lesions at individual level (23, 47, 70) (Table 1). Indeed, Mycoplasma hyopneumoniae (M Hyo), the pathogen commonly responsible for pneumonia (75), is not spread to the lungs via the blood (76), so the pathogenesis of M Hyo—induced enzootic pneumonia is most probably unrelated to tail trauma. Nevertheless, Pandolfi et al. (58) and Valros et al. (26) found an association at animal level between severe tail lesions and pneumonia. In the study by Valros et al. (26), these cases of pneumonia were, however, almost certainly due to some other pathogen as M Hyo is eradicated in Finland.

There are studies showing batch level associations between pleurisy and tail lesions (58, 62, 68). Pleuritis is possibly linked to, for example, a high herd-level prevalence of pigs sero-positive for Actinobacillus pleuropneumoniae (APP) (75). Further, at the level of the individual animal there are strong associations between pleurisy, especially in severe forms [e.g., (70)] and lung abscesses and tail lesions, especially when tail lesions are severe (Table 1). Valros et al. (26) further showed an increase in the occurrence of pleurisy, specifically in pigs with tail lesions that had healed by the time of slaughter. A study including a pathological examination of 56 pigs [selected phenotypically as biters (16), victims of biting (16), controls in biting pen (10), and controls in non-biting pens (14)] from a farm with a tail-biting problem (31) showed that most animals in all these groups (78%) had signs of respiratory inflammations. The severity of these were, however, worse in victims of tail-biting than in the other pigs. Further, Sihvo et al. (69), showed that 9 out of 35 bitten pigs (all 9 had severely bitten tails) had chronic purulent or necrotizing interstitial or bronchopneumonia with (*n* = 7) or without (*n* = 2) abscesses. At least part of these lung lesions were caused by secondary, environmental bacteria, which indicates a possible spread via the venous system from the tail. Other types of lung lesions, such as pulmonary actinobacillosis, and mild lymphocytic interstitial pneumonia were evenly spread between control and bitten animals.

#### Carcass Lesions

There is a well-established association between tail lesions, trimming and condemnation of carcasses (partial or entire) particularly for abscessation at individual pig level (26, 30, 65) (Table 1). Indeed, the lack of an association between carcass condemnation for abscessation and severe tail lesions at individual level in the study by Harley et al. (64) is surprising given that abscessation is the primary reason for carcass condemnation in the Republic of Ireland (24). However, there was an association between mild tail lesions and partial carcass condemnation for abscessation. It is possible that the tail lesions were severe at some stage on-farm and thereby contributed to secondary infection and associated partial carcass condemnation but healed such that they scored mild at the time of slaughter. Infections of the joints (arthritis) and bones (osteomyelitis) were the second most common association between tail lesions and reasons for carcass condemnation/trimming (Table 1). The interrelationship between (poly)arthritis, vertebral osteomyelitis and tail lesions is well-documented [Meijer et al. (67) cited by (30, 63)]. Hence, it is unsurprising that most of these associations were at individual level (23, 25, 60, 66).

## Common Risk Factors for Poor Health and Damaging Behavior

The associations described above could arise from a sharing of risk factors between DB and disease or poor health, even in the absence of any causal relationship. Poor health may take many forms, but the presence of inflammation and/or immune activation is a common feature represented by a centrally organized suite of non-specific “sickness behaviors” (77, 78). These include depression, inactivity, anorexia and sleepiness which evolved to conserve body resources for the high energetic costs of fighting infection (79). Risk factors for poor health span many areas and, in this review, we classify them by the physiological system afflicted e.g., respiratory, enteric and locomotory diseases. They may be infectious factors, such as exposure to pathogens involved in respiratory and digestive disorders, or non-infectious factors including animal predisposition and aspects of management such as climatic environment, biosecurity, housing and diet (75, 80–82). These management factors may increase the level of pathogen exposure, reduce robustness of animals when a challenge occurs, or predispose to physical injury or psychological disturbance. Risk factors for tail-biting are also diverse—decision support tools such as the Husbandry Advisory Tool (HAT) (15) and Schwanzbeiß-Interventions-Programm (SchwIP) (60, 83, 84) list more than 80 different risk factors based on scientific literature. The scientific evidence for risk factors comes from controlled trials [e.g., (85, 86)] or from epidemiological studies (6, 13–15, 57, 84, 87–89). Evidence also comes from commercial experience, e.g., (45, 46, 90, 91). It is beyond the scope of this exercise to review the vast body of scientific evidence on risk factors for DB and particularly disease. Hence, the papers listed are not comprehensive but were selected to illustrate commonality of risk factors for both. Examples of the correspondence of risk factors for DB, or the resulting tail lesions, with risk factors for a variety of health conditions are discussed in the following sections and synopsized in Table 2.

**Table 2:** Studies supporting animal, environment, feeding, housing and management related risk factors shared between health conditions and damaging behavior and the direction of the relationship for specific characteristics.

**Risk factor**	**Characteristic and direction of relationship**	**Damaging behavior**	**Respiratory disease**	**Enteric disease**	**Locomotory disease**	**Other**
Animal	**Sex** males more affected	✓	✓	✓		✓ (mortality, stress and fear)
	**Genotype**	(fast growth rate↑)	✓	~	✓ (slower growth rate↓)	✓ (immune response, disease susceptibility)
	**Birth characteristics** Low birthweight	~ (mediated via ↑ risk in piglets from large litters—see below)	✓	✓	✓	✓ (mortality)
	**Litter characteristics** Large litters	✓			✓ (foot abscess)	
	**Growth characteristics** High nutritional need due to fast or poor growth rate	✓	✓		✓	
	**Coping style and other personality traits** Reactive vs. passive	✓		✓		✓ (heart deviations, neurotransmission, specific and innate immunity)
Environment	**Temperature** Extreme or variable temperatures	✓ (↑ hot)	✓ (↑ cold)	✓ (↑ cold)		
	**Air quality** High ammonia, high humidity, high CO_2_, poor air quality	✓	✓	✓		✓ (ear necrosis, stress, growth rate)
	**Airspeed** Draft	✓	✓	✓		
Feeding	**Composition/Ingredients** Whey and wheat	✓		✓		
	**Diet quality** Mycotoxins	~	✓	✓	-	√ (nephropathy)
	**Diet form** Wet vs. dry Pelleted vs. meal	✓ (wet↑) ✓	✓ (dry ↑)	✓ (dry↑) ✓		
	**Feed delivery**	✓ (non-competitive ↓: ad libitum, timeliness, multiple feed spaces, function of feeding system)	✓ (floor feeding↑)	✓ (restricted feeding↓)	✓ (restricted feeding↓)	
	**Water**	✓ (ease of access↓)	✓ (ease of access↓)	✓ (quality↓, pH)	-	✓ (urolithiasis)
Housing and management factors	**Flooring** Slatted floors	✓	✓ (confounded with no bedding↑)	✓	✓ (low floor quality↑)	
	**Manipulable material/bedding**	✓ (access↓, Δ quality↑)	✓ (access↓)	✓ (access↓)	✓ (straw↓↑, outdoor systems↑)	
	**Farm size**	✓	✓	✓	✓	✓ (death of sows, found dead.-mortality in finishers)
	**Biosecurity/Hygiene**	✓	✓	✓	✓	
	**Stocking density** High density	✓	✓	✓	✓	✓ (Social learning ability↓)
	**Group size** Large groups	✓	✓	✓	✓	
	Regrouping	✓	✓	✓	✓	✓ (suppressed immune function)
	**Weaning age/lactation management** Early weaning Cross-fostering	✓ ✓	✓ ✓	✓ ✓	✓	✓ (risk of death↑)

*✓ Idicates the presence of strong evidence ~ Indicates less compelling or anecdotal evidence*.

### Animal Factors

Susceptibility to disease or poor health and to receipt of tail damage or expression of tail-biting behavior are influenced by factors inherent to the animal itself, as illustrated by the following examples.

#### Sex

Farm and abattoir based studies frequently report that male pigs are more likely to be the recipients of tail-biting (i.e., to have tail lesions) than female pigs irrespective of whether they are castrated (5, 23, 25) or entire (24, 64, 92–94). However, Sinisalo et al. (22) found no difference between the sexes. There is no evidence of a propensity of a particular sex to tail bite. Nevertheless, there was a faster increase in piglets with tail damage in all-female groups compared to piglets in all-male or mixed sex groups post-weaning (94). Furthermore, the duration of tail damage was higher in males in mixed sex groups. As mixed sex groups are the norm in practice, this could explain why more males show tail damage.

The evidence surrounding the propensity for a particular sex to succumb to disease is equally tentative. Male piglets are certainly at higher risk of dying than female piglets (95). They are also more likely to succumb to diseases such as post-weaning multisystemic wasting syndrome (96) and Lundeheim (97) reported that castrates had a higher burden of respiratory diseases than gilts. There is some evidence to suggest pathogenic mechanisms through which males may be more susceptible to disease. Castrated male piglets are more susceptible than females to physiological stress (98, 99) and are more fearful (98, 100, 101) which may increase susceptibility to disease (102). Furthermore, entire male pigs show higher cytokine levels in response to a lipopolysaccharide (LPS) challenge compared to females (103).

#### Genotype

There are a limited number of reports on breed differences in exhibition of DB, [e.g., (104, 105)] as well as in predisposition to be the recipient of tail-biting (5, 22, 33). Generally, it is difficult to separate effects of breed from effects of selection for production traits such as leanness on the propensity to bite or be bitten. Breuer (106) reported that predisposition to tail bite had a heritable component within Landrace pigs and that this predisposition has a genetic correlation with lean tissue growth rate and lack of subcutaneous fatness. Similarly, Ursinus et al. (10) found that gilts with high levels of tail and/or ear biting had a higher genetic potential for several production traits. On the other hand, there is clear evidence that genetic selection for high lean tissue growth rate is associated with changed immune response and increased disease susceptibility (81, 107, 108). Selection for high lean tissue growth rate is also unfavorably associated with leg weakness score linked to osteochondrosis in pigs (109). Finally, Hessing et al. (110) found a strongly significant “litter-effect” on gastric ulceration which might indicate a genetic predisposition.

#### Birth and Litter Characteristics

Tail-biting pigs are often smaller “runt” individuals (23, 111, 112). Munsterhjelm et al. (113) found that performers of “tail in mouth” behavior were smaller at birth. However, there is limited evidence that low birthweight (BW) is a risk factor for DB. Ursinus et al. (10) found no difference in BW between high-tail biters (which also performed more DB directed at other body parts), medium tail-biters and non-tail biters. Similarly, a recent, on-farm study (114) failed to link BW to pig-directed manipulation in the grower/finisher periods. There is some evidence of a predisposing effect of undernutrition during lactation, and therefore low weaning weight, on subsequent manipulatory behavior of weaned piglets whereby the later tail-biting behavior seems related more to growth rate immediately preceding onset of the problem [reviewed by (80)]. This review indicated that DB may be increased in piglets originating from large litters, which would tend to have lower average individual BW, although this is more likely explained by the fact that social competition is greater in large litters. Indeed, Ursinus et al. (10) found that gilts showing high levels of tail-biting originated from larger litters. When it comes to the risk of becoming a victim of biting, Hakansson and Houe (115) recently showed that the risk for tail damage was higher in piglets with a high weaning weight.

There is strong evidence that low BW piglets, more prevalent in large litters, are more susceptible to disease in both the short and longer term. Initially there are long-term dysfunctions in vital organs in low body weight piglets [e.g., gastrointestinal tract—(116)], but they are also less competitive at the udder and ingest less colostrum (117). Piglets with low immunoglobulin G serum concentration have a low survival rate (118), although this might also reflect nutritional and thermal challenges to neonates with low colostrum intake. Calderon Diaz et al. (92) reported that pigs with a low BW (<0.95 kg) were at higher mortality risk throughout the production cycle and were at higher risk of locomotor diseases. In line with this, pigs in large litters had a slightly increased risk of developing foot abscesses, compared with pigs in small litters (119). Also Feldpausch et al. (120) reported a highly increased mortality rate in piglets of low BW. Following on from this, pigs of low BW in larger litters are therefore likely to be at greater risk of death and to be lame prior to slaughter. Furthermore, low BW is a risk factor for respiratory disease (121) and the development of PMWS (96, 122) with the latter study suggesting a threshold <1.3 kg. Calderón Díaz et al. (92) presented a strong positive correlation between birth BW and weaning BW. They found that pigs with a low weaning weight were more likely to have an increased risk of lameness, pleurisy, pericarditis and heart condemnations at slaughter. This is in line with the predisposing effect of undernutrition during lactation on subsequent manipulatory behavior of weaned piglets [reviewed by Prunier et al. (80)], suggesting a common early life background to both health and behavior problems.

#### Growth Characteristics

Clearly, the occurrence of DB in a group negatively affects the growth of the victims. Several studies show lower average daily gain in victims of tail-biting compared to non-victims (22, 61). Similarly, Camerlink et al. (123) reported a poorer average daily gain in pigs that received a lot of oral manipulation (>2% of time), including tail and ear biting and other types of DB. On the other hand, Hakansson and Houe (115), as well as (114) found a greater risk of tail lesions in pigs with a high growth rate before and after weaning. In this case, however, the lesions were not severe, and the weight gain was recorded mainly before the lesions, which implies that high weight gain, or “nutritional need,” predisposes pigs to becoming victims of DB, instead of oral manipulation or tail damage reducing growth rate, as previously suggested. However, it is more likely that the two are not mutually exclusive; while faster growing pigs may be more likely to become victims—they may then have a reduced growth rate if severely bitten. The relationship between growth rate and risk of disease is clearer. Almost all of the common health problems/diseases of pigs are associated with poor growth [e.g., (97)]. A Brazilian study reported an association between lung lesions (pleuritis and embolic pneumonia) and locomotor problems in low body weight pigs at slaughter (59). Similarly, Kuchling et al. (124) reported that expected daily gains reduced significantly for pigs with at least one of the post-mortem findings arthritis, abscess, severe pneumonia, visceralis pleuritis and hepatitis.

The predisposing effect of undernutrition during lactation (80) corresponds to studies showing that pigs exhibiting tail-biting behavior were those that experienced a growth check (111, 112, 125). However, fast growth rates in older pigs may also play a role in the development of DB. At group level, Diana et al. (126) found that pigs with antibiotics in their diet (associated with higher growth rates), were more likely to have tail lesions and performed more ear biting than pigs without. In another study, the fastest growing pens of pigs on a commercial farm were more likely to have ear and tail lesions compared to compromised pigs (127). At individual level, Ursinus et al. (10) reported that gilts showing most biting behavior were also the largest and fastest growing animals. Similarly, Valros et al. (114) reported a high growth rate linked to a higher level of pig-directed manipulation. While pigs that are thriving and growing quickly might be more likely to engage in DB they are almost always less likely to experience health challenges (128). The only condition this does not hold true for is locomotor disorders where selection for fast growth rates is a risk factor for osteochondrosis (109). Furthermore, Quinn et al. (129) reported that gilts fed a diet formulated for slower growth rates (and resulting in reduced average daily gain and lower body weights) had improved locomotory ability and less severe humeral condyle joint lesions compared to gilts on diets designed for fast lean deposition.

#### Coping Style and Other Personality Traits

Prunier et al. (80) defines personality and coping style and elucidates how they may influence the propensity of pigs to perform DB. The effect of coping style appears to be mediated by the degree of environmental enrichment (130, 131), with pigs of a more reactive coping style showing greater frustration (132) more pessimism (133) and more DB with a lack of or loss of, rooting materials. Although, Ursinus et al. (42), found no such link between coping style and DB. Fearfulness is another personality trait relating to tail-biting. Ursinus et al. (134) reported that tail biters spent less time near a bucket introduced in a novel environment, and suggested that tail biters were more fearful. Zupan et al. (34) found the opposite, with tail biters being faster to touch a novel object, lying more and walking less in a novel environment. Linking tail biting to behavior in fear tests is complex as fear-related behaviors in such tests might reflect temporary states rather than stable personality traits. Moreover, tail biting is not consistent, except for those biters that display high levels of tail biting (10, 42). This could relate to the different types of biting identified, with possibly different underlying motivations and risk factors (33).

Pigs show genetic variation in coping style associated with neurotransmission (135). Tail biters had a lower store of serotonin in blood, and a higher blood platelet serotonin velocity. However, this only held for the period in which they were classified as tail biters (134). Valros et al. (136) reported increased serotonin metabolism in the pre-frontal cortex in tail biters and, only in tail biters, a link between central serotonin and tryptophan levels in the blood. However, it is unknown if these differences in serotonin metabolism are temporary states or stable differences between pigs prone to tail bite and those with a lower propensity to tail bite.

Little is known about the link between health and personality in pigs, except that reactive pigs are more susceptible to develop gastric lesions in barren housing than proactive pigs [(137), but see (138), who found no effect] or when housed with other reactive pigs only (139), whereas proactive pigs showed more heart deviations postmortem (139). Several studies point to a difference between proactive and reactive pigs in parameters related to innate and specific immunity [e.g., (101, 140–145)], although the consequences of these differences for disease susceptibility are unknown. According to Kanitz et al. (142), when exposed to stressful situations, the effects of coping style on humoral immunity differs depending on the specific function of the immunoglobulin classes, as proactive pigs may have an increase in IgA concentration but not in IgM. This makes coping style-related differences in disease susceptibility probable.

#### Summary—Animal Factors

There are numerous animal-based risk factors shared between tail-biting and disease but several of these are tentative particularly those relating to coping style and other personality traits (Table 2). Many studies show that male pigs are more likely to be bitten and there is evidence that they are more susceptible to stress (98, 99), disease [PMWS—(96)] and death (95). Furthermore, the limited evidence presented above suggests a propensity of females to bite tails. Hence, there might be sex-based differences in the likelihood of a pig to become a biter or a victim of DB, and to succumb to disease but there is surprisingly little research in either area. Birth weight is a major risk factor for disease (96, 119, 122). However, its effect on tail biting is confounded with litter size whereby piglets from larger litters, being smaller, may be more disposed to tail bite (80). This might suggest that better health and growth rate during lactation reduces the risk of a pig becoming a tail-biter in later life. However, genotypes with generally faster growth rates and lower fatness are more likely to tail bite (10, 106) and groups of pigs with high growth rates appear to be more susceptible to DB [e.g., (10, 127)]. These findings appear to contradict those suggesting that pigs experiencing a growth check are the tail-biting culprits. However, pigs in both cases may have a high “nutritional need/metabolic demand,” either because of their (history of) poor health, poor growth and undernutrition or, conversely, due to a high growth/production level demanding greater nutrients/energy [as per (80)]. This increases the pigs' motivation and competitiveness for access to feed, which in turn can lead to DB (33). Hence, it appears that rather than growth rate *per se*, a high “nutritional need/metabolic demand” is likely the common underlying factor linking health and DB. We elucidate this potential mechanism for a causal link below in section on Elucidating causal mechanisms.

### Environmental Factors

#### Temperature

The effects of temperature on tail biting are contradictory. Smulders et al. (6) found that a higher temperature in the nursery pens was associated with a higher number of pens containing at least one pig with a tail or ear lesion. Similarly, Holling et al. (146) found that a temporary ventilation failure resulting in an increase in temperature was associated with tail-biting. This would suggest that tail biting is higher in the summer. Indeed, Sällvik and Walberg (147) reported that high summer temperatures inside the housing tended to increase tail-biting. However, several epidemiological studies report a higher likelihood of tail lesions in the winter (60, 72, 148). It is likely that fluctuations or extremes of temperature in either direction are risk factors for tail biting and other DB.

Heat stress caused by high temperatures is associated with numerous detrimental physiological changes in the pigs body (149). However, while it undoubtedly leads to poor welfare and poor growth and reproductive performance (150) high temperatures are not directly associated with specific diseases. In this case, there are more implications of cold temperatures. There is an increased incidence of diarrhea in weaned piglets maintained at chronic, moderate cold temperatures (18–20°C) [reports reviewed by (151)]. Armstrong and Cline (152) reported that artificial infection with enterotoxigenic Escherichia coli resulted in higher incidence of diarrhea in newly weaned piglets when exposed to severe cold stress (4–10°C). Temperature fluctuation is also a risk factor—Le Dividich (153) observed a greater incidence of post-weaning diarrhea in piglets kept in a continuous (hourly) fluctuating temperature, 23.5 ± 3°C compared with a constant environment, 23.5 ± 0.5°C. Burrough (154) also identified low temperature as predisposing the development of swine dysentery in endemically infected farms. Many reports of temperature as a risk factor for disease relate to respiratory diseases and Stark (155) cites numerous examples from cross-sectional and longitudinal studies. For example, Geers et al. (156) observed a negative relationship between coughing and air temperature, while the number of treatments for pneumonia was higher for low and highly variable temperatures (157). In a more recent study (75), a mean temperature in the finishing room below 23°C was a risk factor for pleuritis. Similarly, neonatal pigs maintained in a cold environment of 18°C and administered LPS experienced a period of hypothermia coupled with altered endocrine and proinflammatory cytokine responses (158). Additionally, growing pigs kept at high ambient temperature have greater production of heat shock proteins, which are protective during inflammation (159), than those kept at thermoneutrality (160).

#### Air Quality

Whilst linked to temperature, since ventilation rate affects both, several aspects of air quality are also important risk factors for DB and disease. Various authors describe ammonia as the primary noxious gas able to induce stress and consequent DB, including tail-biting (161, 162). Scollo et al. (88) found that high ammonia was the variable showing greatest importance in influencing prevalence of tail lesions with a threshold level of 28 ppm. Ammonia is also a risk factor for liquefaction necrosis (163) and low body weight (164) most likely due to less feeding behavior (165). These authors reported elevated monocyte, lymphocyte and neutrophil counts in weaner pigs exposed to a concentration of 35 ppm using gas exposure chambers, while pigs exposed to 50 ppm showed increased levels of the acute phase protein, haptoglobin. Exposure to ammonia also affects pig activity (164). Done et al. (166) found small pathological changes in the respiratory tract of pigs exposed to concentrations of ammonia representative of commercial conditions (0.6–37.0 ppm). Meanwhile, Stark (155) cited numerous studies implicating aspects of air quality including humidity, gases, bioaerosols and dust. For example, an air exchange rate of >60 m^3^ per h per pig, which would reduce all detrimental air contaminants, had a protective effect on pneumonia (63). Relative humidity (RH) correlated with the number of necessary treatments for pneumonia, but with a lag of 2 weeks (157). High humidity was also a risk factor for ear necrosis syndrome (167). A high mean CO_2_ concentration (>1,600 ppm) in the finishing room was a risk factor for pneumonia (82). This is consistent with previous findings, indicating that high CO_2_ concentrations may have detrimental effects on respiratory tract health (168–171). Furthermore, air quality (a composite score of airflow patterns, NH_3_, CO_2_, RH) is also a risk factor for post-weaning digestive disorders (172).

#### Air Speed/Draughts

Only, Sällvik and Walberg (147) demonstrated that cold air at high airspeed (i.e., draught) tended to increase tail-biting. Exposure to draughts also resulted in more redirected explorative behavior in weaned piglets, including ear biting, and agonistic behavior, especially head knocks with biting (173). In contrast there is good evidence that draughts are a risk factor for disease with increases in coughing, sneezing and diarrhea reported in weaned piglets (174). The setup of the ventilation system seems to be important for respiratory disease. A direct fresh air inlet from outside or from the corridor into the room vs. an indirect air inlet was a risk factor for pneumonia in weaned pigs (75) or for being seropositive to Actinobacillus pleuropneumoniae serovar 3 in finishing pigs (175). Fablet et al. (75) also found that the range of temperatures controlling ventilation rate, which affects how quickly fan speed increases and decreases and may create air draughts at the pig level, was associated with prevalence of pleuritis.

#### Summary—Environmental Factors

The evidence for temperature as a shared risk factor for DB and disease is conflicting. While higher temperatures are risk factors for DB (147), colder temperatures are more frequently cited in relation to disease (Table 2), particularly respiratory disease [e.g., (75)]. Nevertheless, these findings support the well-established requirement to maintain pigs in their thermoneutral zone to optimize welfare, health and performance as recently demonstrated by Chantziaras et al. (176). There is compelling evidence supporting the role of draughts and poor air quality, particularly high ammonia levels as risks for disease. However, it is only in the case of ammonia that this is shared with DB. This is in spite of strong anecdotal and farmer reported evidence of the detrimental effects of draughts (or “cold air pockets”) on tail biting (45).

### Feeding Factors

Risk factors influencing health and DB relate either to the diet itself or to the method of provision. Additionally, both are related to changes in feeding behavior (177, 178). Feed is one of the most contested resources in pigs which is why feeder space is so important, particularly for low-ranking animals. Statements on the number of recommended trough sites vary between different studies. Spoolder et al. (179) recommended fewer than 20 finishing pigs per trough to reduce aggression. Moinard et al. (14) stated the risk of tail-biting increased (OR = 2.7) when five or more grower pigs share one feed space. In the Holmgren and Lundeheim (180) studies, one percent more tail-biting was observed at trough lengths of 30 cm or less per animal. In an experiment with docked piglets, the experimental group with 3.75 piglets per feeder showed no ear and tail lesions, while these injuries occurred in the groups with 5, 6.25, and 7.25, respectively (181). There are no studies demonstrating the effect of feeder space on health of pigs.

#### Composition and Ingredients

Certain dietary ingredients increase the risk of tail-biting. These include whey (87, 180) and wheat (87). Interestingly, a high level of wheat in the diet is also a risk factor for gastric ulcers (182, 183) and non-specific colitis (184, 185). Two old studies report on “whey disease syndrome,” characterized by sudden death associated with acute and extensive hemorrhage into the lumen of the small intestine (186, 187). A more recent study also describes deaths caused by “haemorrhagic bowel syndrome” in pigs fed whey (188). However, this is related to poor feed hygiene practices more than whey feeding *per se*.

The evidence around dietary crude fiber and tail-biting focuses on undocked pigs and is dominated by strong batch and age effects (189, 190). The pigs in these studies were young and therefore generally fed ad-lib and so unlike dry sows (and possibly older pigs—see below) do not experience extreme frustration of feeding/foraging motivation. This is supported by van der Peet-Schwering et al. (191), who found no effect of replacing part of the wheat in the diet with 12–14% of fiber rich feed ingredients on indicators of tail-biting in the nursery. However, they found a lower percentage of grower-finisher pigs with tail damage and with reduced tail length, and a lower number of animals removed to the hospital area on this dietary treatment. It is not surprising that minor increases in dietary fiber level (from 4.2 to 6.6% crude fiber) with minimal enrichment provision did not control tail-biting in undocked finisher pigs on slatted floors (192). Some studies indicate a direct link between ingested fiber and gastric ulcers, e.g., (193). This positive effect is possibly mediated by increased production of saliva (194).

#### Diet Quality

A widely studied aspect of feed quality is contamination with mycotoxins (195–197). There are anecdotal reports of contamination with mycotoxins being associated with tail-biting [e.g., (198)]. High levels of deoxynivalenol were found in feed and sow blood samples in herds where piglets were affected by tail necrosis (199). Similarly, both tail and ear necrosis was observed after a combination of ergotamine, ergocristine, and ergonovine (10 mg/kg of grain) was fed to older pigs (200). Such necrotic lesions may predispose pigs to perform DB but there is no published evidence of a direct link. In contrast, the immunosuppressive effects of mycotoxins and associated implications for disease in pigs is widely studied (201). Specifically, ingestion of mycotoxin-contaminated feed increases the susceptibility to infectious diseases, reactivates chronic infection and decreases vaccine efficacy (202–205). Aflatoxin B1 (AFB1) lowered the incubation period for swine dysentery and prolonged the clinical diarrhea compared to a control group (206). FB1-exposed piglets showed longer shedding of enterotoxigenic Escherichia coli (ETEC) following infection and a lower induction of antigen-specific immune response after oral immunization (207). Similarly, Pósa et al. (208) showed that FB1 aggravated the progression of respiratory disease.

#### Feed Delivery System

The feed delivery system is often confounded with the level, frequency and form of feeding, making it difficult to clearly determine the exact nature of risks. Housing systems with ad lib feeding and multiple feed spaces have less tail-biting compared to restricted and single space feeders (6, 14, 209–212). Similarly, other factors which result in increased feed competition such as dysfunction of the feeding system (15, 90) or deficiency in timeliness of feeding (88) are risk factors for tail-biting.

Stärk (155) identified an adverse influence of floor feeding on respiratory disease (213–215). As mentioned, beneficial effects of restricted feeding for leg health are mediated by slower growth rates [e.g., (129)]. Some studies indicate that strategically restricting feed to piglets in the first few days post-weaning reduces post-weaning diarrhea [e.g., (216)] though others contradict this [e.g., (217)]. Further, the likelihood of oesophago-gastric ulcers at slaughter was higher in pigs from herds with ad *libitum* feeding and herds with automatic feeding (218).

#### Diet Form

Liquid feeding may predispose to tail-biting (14, 17, 87, 219). However, Hunter et al. (209) found that meal or liquid feeding reduced the probability of long-tailed pigs being tail bitten compared to pelleted feed, and Smulders et al. (6) found a higher number of pens with pigs having tail and ear lesions when pigs were fed a dry diet in the growing unit. Differences are possibly linked to differences in feeding frequency, since both Temple et al. (17) and Kallio et al. (87) identified a higher number of smaller meals per day as a risk factor for tail-biting. In agreement, Hessel et al. (220) found that a higher frequency of daily liquid feeds fed to pigs restrictedly resulted in more competition at feeding than a lower feeding frequency.

Use of pelleted compound diets vs. meal was a risk factor for tail-biting in some studies (14, 87, 209). In this respect, it is interesting that pelleting of diets poses a risk for gastric ulcers (183, 221–223) and for non-specific colitis (184, 224, 225). Dry feeding, as opposed to liquid feeding, is a risk factor for Salmonella in pigs (222). It is also generates dust which might increase risk of respiratory diseases (226).

#### Provision of Water

In spite of the obvious importance of water for pig welfare, few studies demonstrate a role in tail-biting. Taylor et al. (15) highlighted a reduced risk of tail-biting when pigs had “good water access” (low ratio of pigs: drinkers, clean drinkers and good water pressure). Further, pig producers ranked “water available to all pigs” as the most important preventive factor for tail-biting (91) or ranked it very high (4th of 20 preventive measures) (46). Inadequate access to water is also a risk factor for respiratory disease (155). Madsen and Kristensen (227) revealed alterations in the circadian rhythm of the drinking behavior of young pigs that is an early indication of health impairment. Furthermore, gastric ulceration was associated with the source of water, with use of bore-hole water being a risk factor (218). This may relate to possible effects of pH, buffering capacity or microbiological quality. Urolithiasis (“stones” in the urinary system) in finishing pigs was partially associated with inadequate provision of drinking water (228).

#### Summary—Feeding Factors

The interrelations between all feeding related risk factors complicates extrapolation of commonality between DB and disease (Table 2). For example, feeding frequency is interrelated with feed form. Hence, while Taylor et al. (15) pointed out that an optimal number of meals/day can minimize tail-biting, the optimal number of meals would need to be determined for all feeding systems and diet forms. The inconsistency is reflected in the effects of liquid feeding, which is a risk factor for DB while dry feeding seems to be a risk factor for certain health problems. Dietary roughage (i.e., fiber from straw or silage) has numerous benefits in terms of facilitating foraging and exploratory behavior, as well as chewing which stimulates saliva production. It also has structural properties that are protective at gut level (193). Not surprisingly there are benefits both to pig behavior, e.g., (229), and health, e.g., (194). However, studies evaluating different levels of dietary fiber show less compelling benefits for tail biting or pig health possibly because growing pigs are generally fed to appetite. The detrimental effects of wheat and whey in the diet and diets in pelleted form are consistent for both DB and disease albeit not widely studied for the former. The same holds for the protective effects of non-competitive feeding systems and easy access to water of high quality. Mycotoxins are the major aspect of diet quality potentially posing risks to pig health and DB but while negative implications for immune function, if not disease itself, are numerous, the evidence for the latter is surprisingly scant.

### Housing and Management Factors

Interrelationships between risk factors associated with housing and management are even more complex than feeding related factors. Such factors comprise confounded and interacting combinations of lactation and social group management, enrichment use, flooring and pen design in addition to all feeding and environmental factors. All of these pose risks for DB and disease.

#### Flooring

Slatted floors are a risk factor for tail-biting (14) with both the presence and the area of slatted flooring implicated (87, 230). This might relate to effects on thermal or physical comfort, air quality or the reduced likelihood of providing bedding. Slatted flooring is also a risk factor for gastric ulcers (48, 221). This could be confounded with the absence of straw bedding, although this is not always the case (231). Stärk (155) identified reports in which slatted floors and poor floor insulation (no bedding) had a negative impact on respiratory health. Sanchez Vasquez et al. (232) demonstrated that part slatted floors compared to solid floors with bedding were a risk factor for enzootic pneumonia-like lesions and pleurisy in the carcass. Aspects of flooring, are also associated with locomotory disorders in finisher pigs (233).

#### Manipulable Material and Use of Bedding

Straw bedding, as well as offering thermal, physical and nutritional benefits, is also a source of enrichment allowing pigs to express foraging and exploratory behaviors. Hence, the absence of this manipulable material is a consistent risk factor for tail-biting (14, 15, 48, 87, 112, 125, 209). Even moderate bedding decreases tail lesions in undocked finisher pigs (234). Smaller amounts of manipulable substrates such as chopped straw (235), light chopped straw and wood shavings (234) or fresh barley straw (236), mushroom compost (237), peat and sawdust (238), grass silage (194), whole-crop barley and pea silage (239), hessian fabric (10) or freshly cut wood (85) also reduces penmate-manipulations/tail and ear biting. Buijs and Muns (240) summarize the non-straw enrichment material benefits for tail-biting in more detail. It is also worth mentioning that a change in the quality of enrichment between the production stages is a risk factor for tail-biting (15).

There are some concerns for pig health associated with straw provided as bedding or enrichment due to the potential presence of mycotoxins (241). Pigs are susceptible to several types of mycotoxins which have a detrimental impact on immunocompetance (201, 242) if not pig health. Other authors show health challenges for pigs on deep bedding associated with poorer hygiene, including PMWS susceptibility and respiratory/enteric health though there were positive effects on leg health (48). Provision of straw or other fibrous material also reduces the risk of gastric ulceration (194, 221, 243–246) and other stomach and intestinal disorders (247). This could be due to the beneficial effect of fiber ingestion *per se* (193, 246). However, Gottardo et al. (231) found the provision of other forms of environmental enrichment (hanging chains, plastic objects) also decreased the risk of gastric ulceration on farms that did not provide bedding. There is also evidence of a protective effect of environmental enrichment against other disease challenges. For example, pigs reared under enriched conditions exhibited fewer days of diarrhea after weaning (248) while those raised with a combination of social and environmental enrichment factors had a faster viral clearance and developed fewer and less severe lung lesions after an artificial disease challenge (2).

#### Farm Size

Larger farm size is a consistent risk for disease and DB (14, 83, 87, 88, 249) with Moinard et al. (14) and Scollo et al. (88) also identifying more pigs per stockperson as a risk for tail-biting.

Stärk (155) cites numerous studies in which herd size was a risk for respiratory disease, with subsequent studies also highlighting a specific risk for pleurisy (250, 251). Ramis et al. (252) suggested that an increase in gastric ulceration in pigs from large farms might be due to increased infection pressure from other diseases, but there are implications of many other non-infectious factors associated with herd size. Goldberg et al. (253) see large herd size as a significant risk factor for mortality in sows, also supported by Bergman et al. (254). Further, Munsterhjelm et al. (177) found an association between farm size and “found dead” mortality in finishers.

#### Biosecurity/Hygiene

In epidemiological studies both Smulders et al. (6) and Pandolfi et al. (58) reported links between poor biosecurity practices and DB/tail lesions. For example, the former authors found that the absence of footbaths was a risk factor for tail and ear biting. Furthermore, tail-docked pigs subjected to low sanitary conditions showed increased ear-biting behavior and damage to ears in comparison to unchallenged animals, although this effect was diet dependent (43).

Good hygiene and health management can prevent, as well as interrupt, the spread of infection. Generally, poor biosecurity practices pose a greater risk of disease [e.g., (58, 255)]. Pen hygiene is particularly important for enteric disease. Madec et al. (172) showed that pen hygiene status on arrival posed a risk for post-weaning digestive disorders. Non-hygienic husbandry, inadequate quarantine and biosafety measures are also risk factors for PMWS (256–258). Pen hygiene predisposes development of swine dysentery in endemically infected farms (154). A Polish study reported that farms with an all-in/all-out hygiene policy (AIAO) had a significantly lower prevalence of Lawsonia intracellularis (259). In addition, the odds of having a Salmonella-positive sample was 3.9 times lower in farms practicing AIAO (222). The importance of biosecurity, including animal purchasing policy, AIAO management and hygiene in the occurrence of respiratory disease are well-known (253, 260, 261) [see also (155) for review]. Finally, pen hygiene influences prevalence of locomotory disease through increased risk of infectious agents, but also softening of the claw in wet conditions (262) and increased risk of slipping and injury on soiled floors (263).

#### Stocking Density

As stocking density is the combination of pen size and number of pigs per pen, either factor separately or in combination can influence its effect. Most studies, both epidemiological and experimental, report more tail-biting in pens with higher stocking densities (14, 88, 177, 219). For example, weaner pigs with lower stocking density (<38 kg/m^2^) had a lower prevalence of tail lesions than pigs in pens with a stocking density ≥38 kg/m^2^ (84).

High stocking density also increases the risk of many health problems, including non-specific colitis (224), swine dysentery (154) and respiratory disease (155), clinical leg weakness and claw disorders (264). Tuovinen et al. (265) estimated an odds ratio of partial carcass condemnation of 4.2 for a decrease of the total pen area per pig by 0.1 m^2^. Stocking density influences not just floor space, but also three-dimensional space. More than 3.5 m^3^ of air volume per pig was preventive for pleurisy (63).

#### Group Size and Composition

Group size, although sometimes confounded with stocking density, can influence disease transmission and the scope for DB. Holmgren and Lundeheim (180) found that an increase of one pig to the group increased the prevalence of tail-biting by +0.2% with long-tailed pigs, and suggested that this was a consequence of an increase in the number of potential victim pigs. Kallio et al. (87) also found more tail-biting when finishing group size was >9 pigs. Reducing the size of the group from 15 to 12 finisher pigs reduced tail-biting despite a tendency for more tail-directed behavior (266, 267).

Madec et al. (172) showed that larger group sizes (>23 pigs/pen) increased the risk for post-weaning digestive disorders, whilst the risk of PMWSwas greater with large pens (258) or more pigs per pen (122). Higher group size is also linked to risk of non-specific colitis (268) and to respiratory disorders (63, 168). Furthermore, pigs housed in large groups had poorer leg health compared with pigs housed in small groups (262, 269).

#### Regrouping

Friend et al. (270) found more tail lesions when more litters were regrouped. In agreement, Gruempel et al. (84) reported a lower prevalence of tail lesions in pens where there was less regrouping at weaning. Interestingly, Arey (271) reported an outbreak of tail-biting in pigs after regrouping in new pens with twice as much space as before. Regrouping unfamiliar pigs is a cause of stress and de Groot (272) suggested that this was responsible for suppressing the immune response to a viral vaccine. Regrouping is a risk factor for PMWS (256, 258) and for locomotory disorders (273). It is also a possible risk factor for gastric ulceration (110, 231) while more frequent movement of animals is a risk factor for respiratory disease (213, 214, 274).

#### Lactation Management and Weaning Age

Moinard et al. (14) found a higher incidence of tail-biting in farms where cross- fostering was practiced compared to farms with no cross-fostering. However, it was uncertain whether cross-fostering contributed directly to later likelihood of tail-biting occurrence or whether this association was related to a common causal factor (for example, herd size or litter size). Hakansson and Houe (115) reported cross-fostering was associated with a higher probability of tail damage post-weaning. In contrast, Calderón Diaz et al. (275) found no influence of early or late cross-fostering on the occurrence of tail lesions. However, pigs from fostered litters were more at risk for death and euthanasia, with severe tail lesions being one of the reasons for euthanasia. Furthermore, cross-fostering placed pigs at greater risk of subsequent pericarditis and heart condemnations (275). A high level of cross-fostering is also a risk factor for PMWS (258).

In pens of weaners with high stocking density, prevalence of tail lesions was lower if total piglet losses were <18 % (12%, *n* = 45) and higher, if suckling piglet losses were ≥18% (26%, *n* = 116) (84). This might suggest that better health during lactation reduces later tail-biting risk.

In general, piglets weaned later are more developed and perform less manipulatory behavior after weaning (276–279), which might reduce DB. However, available data suggest that age at weaning (one to 6 weeks) had no clear influence on tail and ear biting in growing pigs even though manipulatory behavior was at least transiently increased in the piglets weaned at the earlier ages (one to 2 weeks compared to 4 weeks, but also 4 weeks compared to 6 weeks) [reviewed by (80)].

Early weaning stress (16 and 18 days of age vs. 20 days of age) resulted in immediate and long-term deleterious effects on intestinal defense mechanisms (280). Early weaning age [e.g., <26.5 days) increases the risk of developing PMWS (172, 281). Piglet age at weaning was also a risk factor for respiratory disease (121, 282). Moreover, the risk of lameness for pigs weaned at a younger age was higher (275).

#### Weaning Stress

The weaning period is associated with a lot of stress for piglets including removal from the sow, dietary changes, regrouping of pigs from different barns or even farms as well as adapting to a new environment. Indirect evidence suggests that increased weaning stress is associated with more DB. Treatments designed to facilitate the weaning transition, such as perinatal flavor conditioning (283, 284) or playful foraging during the lactation period (285) not only improved growth and feed intake post-weaning, but also reduced DB (248, 284) or number of pigs with ear or tail damage (285).

At weaning, histological changes in the small intestine have a negative effect on the immune system and lead to intestinal dysfunction which favors post-weaning diarrhea (286) and is aggravated by reduced feed intake. Hence, post-weaning alterations in gut functioning can increase disease susceptibility and mortality rates [reviewed by (287)].

#### Summary—Housing and Management Factors

Animal, feeding and environmental risk factors have a direct effect on the animal. In contrast, some of the risk factors associated with housing and management influence the animal, and therefore DB and disease, indirectly. For example, effects of farm size and biosecurity are probably mediated by related issues such as staffing level, hygiene practices and farm layout (288). Large farms are also more likely to have large group sizes. These in turn are often associated with more regrouping of pigs, although poor matching of pen sizes or uneven growth can also be reasons for repeated regrouping (128, 289). In any case, the direct detrimental effects on tail-biting and health of frequent regrouping, large group size and high stocking densities are consistent (Table 2). The same holds for the protective effects of solid floors and provision of manipulable materials. The benefits of bedding for tail biting and disease likely relate to better thermal and physical comfort and the fact that straw also acts a source of roughage. Indeed, the fact that pigs on slatted flooring do not have bedding and therefore a source of roughage, is a major reason why slatted floors are a risk factor for tail-biting. Straw bedding has some detrimental impacts on pig health but these are likely mediated by management factors such as poor hygiene and use of poor quality straw. Clearly, any housing and management practices that minimize weaning stress can simultaneously improve pig health and welfare and thereby the risk of disease and tail biting. Higher age at weaning is protective for pig health but while there are indications it is also protective for DB, the evidence is limited. Finally, aspects of lactation management such as cross-fostering are increasingly associated with later tail-biting (14) and poor health. However, it is uncertain whether cross-fostering contributes directly to later likelihood of problems, or whether the association is related to a common causal factor (for example, herd size or litter size).

## Evidence of Possible Causality in the Link Between Damaging Behavior and Disease

Previously, we outlined the risk factors shared between disease and DB and discussed how they can mediate tail biting and poor health. It is possible that apparent associations between DB and poor health are a result of independent responses to such risk factors, but there is also evidence of direct links between DB and poor health. In this section, we explore the rather limited evidence for a two-way relationship, suggestive of causality, between these two problems. Despite the lack of data, the presence of either of these problems on a farm facilitates the development of the other, as they are both stressors (6, 38, 290).

### Disease—A Way to Spread Damaging Behavior

The best known example of disease stimulating DB is the link between exudative dermatitis or “greasy pig disease” and ear biting behavior, e.g., (50, 291, 292) cited by Schroder-Petersen and Simonsen (38). In general, these authors postulate that the associated lesions on the ears make them attractive to other pigs and encourage pigs to start biting the affected ears. Similarly, Clegg et al. (293) suggest that lesions on the body, and particularly on ears, is what leads to biting by other animals in the same pen. However, this hypothesis is untested.

### Damaging Behavior—A Way to Spread Disease

Another hypothesis is that pig behavior causes trauma to the ears providing a point of entry for bacteria to set up infection and subsequently ear necrosis [(294, 295) cited by (167)]. Young piglets already perform seemingly non-injurious ear directed behavior in commercial systems [e.g., (296–298)]. Such behavior could be a precursor to DB and therefore causative in the development of ear necrosis. Indeed, most of the bacteria implicated in ear necrosis cannot proliferate in the absence of an initial trauma to the skin of the ear (299). While aggressive behaviors do not fit with the definition of DB employed in this review, the associated injuries to the face and ears arising from such behavior at weaning, also act as entry points for bacteria and thereby disease (294, 300). Similarly, Mirt (299) considers “playing with the tips of the ears,” and “thrusting at each other's flanks” as being enough to cause trauma to provide a point of entry of bacteria.

Damaging behavior can act as the vector of pathogens. Karlsson et al. (301) and Clegg et al. (293) suggest a possible infection route through biting illustrated by the relationship between the presence of Treponema spp. in porcine skin lesions and in gingiva. Thus, biters can infect their victims. Once tail lesions are infected, systemic spread of infection may occur mainly via the venous route targeting the lungs, and to a lesser extent via lymphatic spread (69) as suggested by Schroder-Petersen and Simonsen (38). Spread of pathogens from the bitten tail can also lead to the formation of abscesses, especially on the back area of carcasses (25) or to pyaemia (68) and to embolic pneumonia (59, 65).

Finally, tail-biting can be a transmission route for trichinosis by the ingestion of infected swine flesh (302, 303).

## Elucidating Causal Mechanisms

This review indicates strongly that tail-biting and poor health are linked. A possible link between other forms of DB and poor health is hardly investigated, although there are indications that ear lesions arising from ear biting may lead to ear necrosis (299). However, ear biting differs from tail-biting in that pigs may damage each other's ears during aggressive interactions (304, 305) as well as through DB (37). Links between tail biting and disease are reflected in the positive correlations between tail lesions and (postmortem) signs of (particularly respiratory) disease at farm and batch level. In spite of issues of confounding and interrelationships, the numerous examples of commonality of risk factors between tail biting and disease provide the first line of evidence for possible causality. This means that efforts to address non-infectious risks for certain diseases will simultaneously reduce the risk of DB on the farm and vice versa. This will thereby contribute to more (cost) effective prevention strategies for both (306). Nevertheless, the sharing of risk factors also complicates elucidating causality experimentally. Proving causal relationships is only possible in controlled experimental studies but such studies usually only concentrate on one or a few risk factors simultaneously and therefore fail to address the multifactorial nature of the problem.

The evidence for causal relationships between lesions arising from tail-biting and disease, is limited. However, the findings of this review support two likely causal relationships. The first is that generalized poor health (e.g., enzootic pneumonia) on farms poses an increased risk of pigs performing DB. The second involves tail-biting causing poor health, both indirectly, through pathogens entering the body via the (tail) lesion followed by systemic spread of infection through the body, or directly, whereby biters infect victims. These causal mechanisms are supported by some actual proof of health problems increasing the risk of DB (51), and of DB increasing the risk of poor health [e.g., (69)].

As well as infection caused by disease itself, many of the non-infectious risk factors which are shared between poor health and tail-biting induce the production of cytokines by the innate immune system (53). Indeed this immune activation by non-infectious as well as infectious risk factors (307–309) could explain the seemingly cumulative effect of risk factors when it comes to outbreaks of tail-biting (8, 35). Studies in humans (78, 310–312) and rodents (313–315) indicate a causal role for pro-inflammatory cytokines in the deterioration in mood experienced during some types of illness, and their putative role in clinical depression for some patient subtypes is under investigation. These cytokine-mediated changes in mood could lower the threshold to show DB toward conspecifics. Experimental studies tested the effects of immune activation (by means of LPS injection) on behavior in gilts housed in triplets (52). In addition, there are correlations between cytokines and behaviors related to tail-biting in intact boars (51). LPS seemed to increase tail-biting related behavior in the gilts after the sickness symptoms abated, and there was a correlation between different cytokines and tail-biting related behavior in the intact boars. Nordgreen et al. (53) discussed these findings and the putative mechanisms linking cytokines to a change in behavior.

Vaccination stimulates the immune system in a similar way to a disease challenge. In laying hens, there is evidence that vaccination may stimulate feather pecking because of activation of the immune system (316). In pigs the limited evidence is to the contrary, i.e., that vaccination might reduce DB. For example, Almond and Bilkei (317) found that an oral vaccine against Lawsonia intracellularis resulted in less social stress and what they described as “cannibalism-related waste” compared to unvaccinated pigs. However, the paper did not investigate effects of the vaccine on DB. Similarly, other authors report that vaccination against PCV2 was effective in the reduction of ear necrosis syndrome, but we do not know if this was mediated by a reduction in DB (318, 319).

We describe numerous farm and abattoir based studies showing associations between tail and respiratory lesions reflecting infection with enzootic pneumonia. This disease is associated with generally poor health and therefore a generalized inflammatory response (68). Based on the putative mechanism outlined by Nordgreen et al. (53), the presence of such a respiratory disease likely increases the risk of pigs with a high disease burden to perform DB. This could conceivably lead to a circular relationship between poor health and DB, whereby all pigs on such a farm would have a generalized inflammation making (some of) them more likely to tail bite. In doing so, a strong inflammatory/acute phase response arising from tail damage would be induced in their already compromised (inflammatory wise) victims (29). In support of this, in some studies pigs act as both biters and victims (134). Clearly, this complicates ascertaining “what comes first,” tail-biting or illness.

Suboptimal nutrition could be an important link between generalized poor health on a farm, the associated activation of inflammatory processes and increased risk of DB. Several reviews point to suboptimal nutrition as a risk factor for DB [e.g., (33, 320)]. Dietary deficiencies enhance foraging-related exploratory behavior (321, 322) which, if directed at another pig might increase the risk of DB. Moreover, some dietary deficiencies may increase the attractiveness of blood [protein deficiency: (323); salt deficiency: (39, 40)], which could act as an incentive to sustain DB. Several mechanisms could play a role in the link between nutrition, poor health and DB. Firstly, there are negative effects of generalized poor health on the ingestion and processing of nutrients. At its simplest and as discussed earlier, poor health can in this way cause growth retardation, which is a potential cause of tail-biting (112, 125). Secondly, health problems also alter the requirements for specific nutrients, like protein or essential amino acids, which could potentially lead to dietary deficiencies, in particular if the diet contained just sufficient amounts of such nutrients for a healthy, unstressed state. The essential amino acid tryptophan is the precursor of serotonin, a neurotransmitter that is related with tail-biting (134) and its metabolism is associated with inflammatory processes. Part of the inflammatory response is to increase tryptophan catabolism (324) so that tryptophan is not available for pathogens (325). Hence, a bout of illness could spark a tryptophan deficiency, potentially leading to DB. Indeed, tryptophan supplementation decreases ear and tail-biting (326). Also low dietary protein levels increase the occurrence of DB like ear and tail-biting and other manipulation directed to pen mates (43). The same study also reported a diet-dependent effect of poor sanitary conditions on ear biting. Supplementing the diet with a 20% increase of amino acids important in the inflammatory response, i.e., tryptophan, threonine and methionine, reduced ear biting only in the pigs housed in poor sanitary conditions. In line with this, Pastorelli et al. (327) showed decreased growth and increased activity and trough-related exploration in response to a reduction in diet quality in pigs kept in unhygienic conditions. There was no such effect in pigs in good sanitary conditions, suggesting that immune-stimulated pigs may have greater susceptibility to specific nutrient deficiencies. Thus, there are clear examples of nutritional deficits influencing DB, suggesting that poor health could enhance DB via its effect on the metabolic status of pigs. Taken together this suggests that there may be scope for reducing DB when pigs are challenged via customized nutritional programs.

Linked to suboptimal nutrition, “gastrointestinal discomfort” is a risk factor for tail-biting (328). Indeed, Van den Berg et al. (329) reported a higher prevalence of gastric lesions in pigs from herds with clinical signs of tail-biting. However, causation was not proven and shared underlying risk factors such as diet form and ingredients, genotype and coping style cannot be ruled out. Perhaps pigs that suffer from gastric lesions and ulcers may increase their chewing behavior in an effort to increase saliva production and stomach pH and thereby reduce gastrointestinal discomfort, and this manifests in increased tail or ear biting (328). However, Munsterhjelm et al. (31) did not find a difference between tail biters and non-biters in gastric lesions. Furthermore, Palander et al. (330) hypothesized that tail biters might suffer from a poor ability to digest and absorb nutrients. Although there were differences in gut morphology between pens with and without tail-biting, blood minerals and amino acids, no signs of malabsorption was found in tail biters. Finally, a role of the “brain-gut-microbiota axis” in the occurrence of tail-biting is proposed (328) and there is early research suggesting a link between gut microbiota and DB (tail-biting) in pigs (331).

This review provides clear evidence of associations between tail lesions and pathological lesions indicative of secondary infection (abscessation, bone and joint infections) in the individual animal. Further, in individual victims of tail-biting, especially if the tail lesion is severe, there is an increased risk of secondary lesions in the lungs, or complications of lung inflammation. These findings support the theory of venous or cerebrospinal fluid spread of infection/pathogens from the tail lesion site beyond the coccygeal region (69). There is no supporting experiment evidence and indeed, it would pose considerable ethical challenges to do so (332). Logistically the low prevalence of abscessation in slaughter pigs means that, even in epidemiological abattoir-based studies, it is difficult to detect enough cases to generate support for the link. The absence of associations between lesions associated with secondary infection and tail lesions at batch level (62, 74) provides further evidence of a causal link. Should an association at batch level exist, it would suggest that the portal entry of the infectious agent was not necessarily the tail. Indeed Martinez et al. (66) proposed that an environment predisposing pigs to other injuries could have provided a port of entry for pathogens leading to abscess formation as a possible explanation for the lack of an association between tail lesions and abscessation in individual pigs in their study. In that study, only growth-retarded slaughter pigs were involved and there was a high prevalence of abscessation relative to pigs affected by tail lesions (only 0.25% of pigs affected). The authors also suggested that another explanation for the lack of association might be the fact that the pigs had tail lesions that had healed by the time of slaughter. Marques et al. (59) also noted this complication. In explaining the origin of some spinal abscesses in the absence of obvious tail damage, Huey (30) suggested that, since the development of spinal cord abscesses takes several weeks or even months, the original tail lesion could disappear before slaughter. Indeed Valros et al. (26) showed increased pathologies in slaughter pigs with fully healed tail lesions.

Antibiotics are used to treat pigs with tail lesions. Studies from Finland, where antibiotic use is low and tails are undocked, show that tail lesions are one of the most common reasons for treatment of individual pigs (333, 334). Therefore, associations between tail lesions and abscessation raise questions about the effectiveness of the antibiotics or treatment protocols. The Norwegian Medicines Agency recommends benzylpenicillin procaine to treat tail wounds (335). Penicillins reach sufficient concentrations in soft tissue and are therefore effective against anaerobic bacteria, which cause abscesses (336). Hence, if administered soon enough this active agent should kill the bacteria before abscesses form. However, delayed treatment means that secondary infections encapsulate locally within the body such that the antibiotic cannot reach them. Indeed, penetration of an antibiotic into an encapsulated purulent lesion is limited and highly dependent on the degree of abscess maturation (337). Spinal cord abscesses may take several weeks or even months to develop (30). Assuming some sort of intervention is made to protect a tail bitten pig from further damage [see (338)], cutaneous healing of tail injuries occurs over 3–7 days (339). Therefore, it is plausible that treatment of pigs with antibiotics stops once the original tail lesion heals. This might be too soon to prevent the development of abscesses. As far as we are aware, there are no studies showing the effectiveness of antibiotics in reducing the risk for secondary infections in relation to tail-biting. Neither are there studies defining how serious a tail lesion should be to benefit from treatment with antimicrobials, or which treatment protocols are most efficient. Given the risk of secondary infection to the welfare of the pig, to food safety and the threat of antimicrobial resistance, arising from mis/over use of medications there is clearly a need for research to inform guidance on the sustainable use of antimicrobials in the treatment of tail lesions.

## Conclusions

Sharing of common risk factors and correlations between poor health and DB and sharing of risk factors is evidence of a clear link at individual and batch/farm level. Their circular relationship, with some evidence of two-way causal mechanisms, makes it difficult to understand the complex underlying mechanisms linking poor health to DB. Nevertheless, the undeniable links offer hope of simultaneous progress on two of the main challenges to the sustainability of pig production, namely, growing public concerns for pig welfare and the threat of antimicrobial resistance arising from mis/over use of antibiotics. The myriad of links between tail-biting, one of the major threats to pig welfare and poor health, the major cause of antimicrobial use, means that efforts to address non-infectious risks for certain diseases will simultaneously reduce the risk of DB and vice versa. At a practical level, the findings have two important implications for the renewed efforts to stop tail docking pigs in the EU. Firstly, the increased risk of tail-biting in long tailed pigs, at least in the interim, means there is an urgent need for protocols for antibiotic use in bitten pigs. Secondly, farmers need to focus on overcoming disease challenges, as well as on reducing common risk factors for disease and tail-biting if they are to raise undocked pigs successfully.

## Author Contributions

All authors listed have made a substantial, direct, and intellectual contribution to the work and approved it for publication.

## Conflict of Interest

The authors declare that the research was conducted in the absence of any commercial or financial relationships that could be construed as a potential conflict of interest. The handling editor declared a past co-authorship with one of the authors, SE.

## Publisher's Note

All claims expressed in this article are solely those of the authors and do not necessarily represent those of their affiliated organizations, or those of the publisher, the editors and the reviewers. Any product that may be evaluated in this article, or claim that may be made by its manufacturer, is not guaranteed or endorsed by the publisher.
